# Gut Microbiome Disruption in Shelter Cats with Feline Panleukopenia: Virome Co-Detection and Enteric Dysbiosis

**DOI:** 10.3390/biology15131087

**Published:** 2026-07-06

**Authors:** David Purec, Vlad Iorgoni, Ionica Iancu, János Dégi, Corina Pascu, Luminița Costinar, Corina Badea, Alexandru Gligor, Paula Nistor, Alexandru Udrea, Ioan Cristian Dreghiciu, Viorel Herman

**Affiliations:** 1 Department of Infectious Diseases and Preventive Medicine, Faculty of Veterinary Medicine, University of Life Sciences “King Mihai I”, 300645 Timisoara, Romania; david.purec.fmv@usvt.ro (D.P.); ionica.iancu@usvt.ro (I.I.); janosdegi@usvt.ro (J.D.); corinapascu@usvt.ro (C.P.); luminita.costinar@usvt.ro (L.C.); corina.badea@usvt.ro (C.B.); alexandru.gligor@usvt.ro (A.G.); paula.nistor@usvt.ro (P.N.); alexandru.udrea.iosud@usvt.ro (A.U.); viorel.herman@fmvt.ro (V.H.); 2 Department of Parasitology, University of Life Sciences “King Mihai I”, 300645 Timisoara, Romania; cristian.dreghiciu@usvt.ro; 3 Academy of Romanian Scientists, Str. Ilfov, Nr. 3, Sector 5, 50044 Bucharest, Romania

**Keywords:** parvoviral enteritis, kittens, intestinal ecosystem, shelter epidemiology, diagnostic interpretation, vaccine-associated shedding, microbial reassembly, longitudinal sampling

## Abstract

Feline panleukopenia virus (FPV) causes severe enteric and systemic disease in cats, with particular importance in shelter environments where susceptible kittens, high population turnover, environmental contamination, and variable vaccination histories increase infection pressure. Recent virome and microbiome studies suggest that FPV-associated enteritis may occur within a broader context of viral co-detection and intestinal microbial disturbance, but direct FPV-specific bacteriome evidence remains limited. This review aims to synthesize current evidence on FPV-associated enteritis in shelter cats by integrating viral pathogenesis, diagnostic interpretation, enteric virome co-detection, gut dysbiosis, recovery dynamics, and intervention-related ecological effects. The literature was organized using an evidence-tier framework that distinguishes direct FPV/feline panleukopenia evidence from feline enteric microbiome proxy evidence and broader comparative or mechanistic microbiome studies. This approach was used to define the limits of inference and to separate evidence-supported conclusions from hypothesis-generating ecological models. Feline panleukopenia in shelter cats should be interpreted not only as an individual viral infection, but also as an ecological process shaped by host susceptibility, shelter exposure, diagnostic complexity, viral co-detection, and microbial community disturbance. Current evidence supports a cautious framework in which virome co-detection and dysbiosis-associated patterns are not treated as direct proof of causation. Future longitudinal, context-controlled, and multi-layer studies integrating validated FPV diagnostics, virome and bacteriome profiling, clinical metadata, treatment records, and functional endpoints are needed to clarify the biological and clinical significance of gut ecosystem disruption in feline panleukopenia.

## Simple Summary

Feline panleukopenia is a severe viral disease that mainly affects young and unvaccinated cats, especially those living in shelters where many animals share the same environment. This review examines not only the virus itself, but also how the disease may disrupt the natural balance of microorganisms living in the intestine, including bacteria and other viruses. Current evidence suggests that shelter conditions, stress, crowding, frequent animal turnover, and medical treatments may all influence these intestinal microbial communities during illness and recovery. However, most existing studies are limited and cannot yet determine whether these microbial changes directly worsen the disease or are simply consequences of intestinal damage. The review also highlights important diagnostic challenges, including the difficulty of distinguishing natural infection from temporary viral shedding after vaccination. In addition, it discusses how treatments such as antibiotics, diet changes, probiotics, and supportive care may influence recovery by altering the intestinal ecosystem. The article concludes that future research should focus on long-term studies that follow cats from shelter intake through recovery, while combining clinical information with detailed analyses of intestinal microorganisms. Such work may improve disease control, treatment strategies, and long-term health outcomes for shelter cats.

## 1. Introduction

Viral enteritis in cats is a heterogeneous clinical and ecological condition involving multiple viral agents, host factors, and environmental contexts [1,2,3,4,5]. Feline panleukopenia (FPL), caused by feline panleukopenia virus (FPV), remains a severe and epidemiologically important disease, especially in high-density populations such as animal shelters [2,6,7,8,9]. Shelters are characterized by continuous intake of susceptible cats, frequent population turnover, complex contact networks, environmental contamination, and variable vaccination status, all of which may favor viral persistence and transmission [7,8,10]. Although FPV outbreaks may also occur in household, breeding, or clinical settings, this review focuses on animal shelters because shelter populations provide a well-documented context in which repeated exposure, environmental viral persistence, and outbreak risk converge [7,8,10,11].

**Figure 1:**
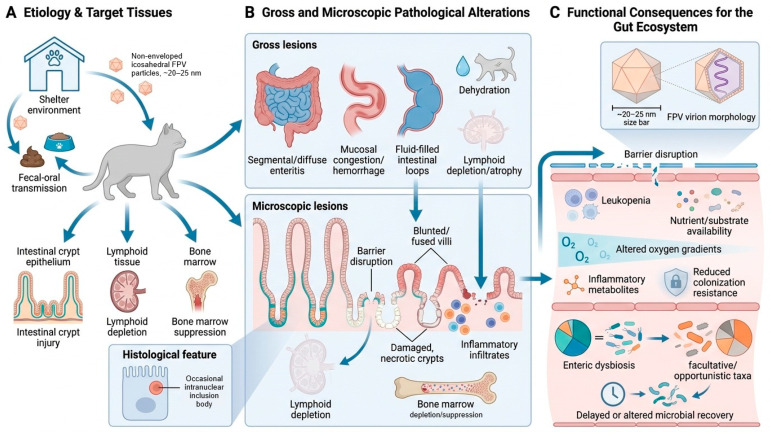
Pathological basis of FPV-associated gut ecosystem disruption. (**A**) Etiology and target tissues, showing fecal–oral transmission in the shelter environment, FPV morphology, and the main target tissues: intestinal crypt epithelium, lymphoid tissue, and bone marrow. (**B**) Gross and microscopic pathological alterations, including enteritis, mucosal congestion or hemorrhage, fluid-filled intestinal loops, dehydration, lymphoid depletion, crypt injury or necrosis, villous blunting or fusion, inflammatory infiltrates, bone marrow depletion or suppression, and occasional intranuclear inclusion bodies. (**C**) Functional consequences for the gut ecosystem, including barrier disruption, leukopenia, altered nutrient and oxygen gradients, reduced colonization resistance, enteric dysbiosis, opportunistic expansion, and delayed or altered microbial recovery. Colors and arrows are schematic and indicate the progression from FPV exposure and tissue injury toward gut ecosystem disruption.

Feline Panleukopenia Virus (FPV) is a small, non-enveloped, icosahedral parvovirus of approximately 20–25 nm in diameter, with a linear single-stranded DNA genome enclosed within a protein capsid. Its lack of a lipid envelope contributes to environmental stability and resistance to many routine disinfectants [3,5,6,12]. The virus has a marked tropism for rapidly dividing cells, particularly intestinal crypt epithelium, lymphoid tissues, and bone marrow [1,2,6]. Infection may result in crypt epithelial necrosis, impaired epithelial renewal, villous collapse or atrophy, mucosal barrier disruption, lymphoid depletion, bone marrow suppression, and leukopenia [1,2,6,13,14]. Occasional intranuclear inclusion bodies may be observed in infected crypt epithelial cells, although they are not consistently detected in routine histological sections [6,15]. These pathological changes, summarized schematically in Figure 1, provide the biological basis for interpreting FPV enteritis as an intestinal ecosystem disturbance rather than only a viral detection event.

In this review, dysbiosis refers to disruption in the composition, diversity, or function of the intestinal microbial community, whereas symbiosis refers to a balanced host–microbiome relationship supporting barrier function, immune regulation, and metabolic stability [16,17]. Prebiotics are substrates selectively used by host-associated microorganisms [18], whereas probiotics are live microorganisms administered with the aim of conferring a health benefit [19]. These definitions are important because dysbiosis and symbiosis describe microbial or host–microbiome states, whereas prebiotics and probiotics describe potential intervention strategies [16,17,18,19].

Metagenomic and targeted virome studies in shelter populations have shown that individual cats may carry multiple enteric viruses, often without clinical disease [20,21,22]. Therefore, viral detection alone should not be interpreted as direct evidence of pathogenesis. Co-detected viruses may represent shared shelter exposure, subclinical carriage, secondary passengers, or potential modifiers of disease severity, but their pathogenic role requires clinical, temporal, and diagnostic correlation [20,21,22,23]. Studies comparing cats with feline panleukopenia to shelter controls have reported increased frequencies of viral co-detection in affected animals, although these cross-sectional data cannot resolve temporal sequence or causality [22,23].

The interpretation of FPV infection is further complicated by diagnostic and biological confounders, including vaccine-associated shedding, asymptomatic viral carriage, variable assay performance, and genetic diversity among circulating parvoviruses [23,24,25,26,27,28]. Molecular and antigen-based assays may detect vaccine-derived viral shedding in recently immunized cats, particularly within defined post-vaccination windows, introducing a risk of false-positive classification in the absence of compatible clinical disease [24,25,27]. In shelter settings, prospective diagnostic work has shown that point-of-care antigen testing and qPCR do not provide fully interchangeable information and that weak-positive antigen results require cautious interpretation [27]. Naturally infected cats may also show genetic complexity and multiple parvovirus infections, adding further interpretive difficulty beyond simple positive/negative classification [28]. These factors support a framework in which FPV detection is interpreted together with clinical presentation, vaccination history, assay type, viral load when available, and epidemiological context.

In parallel with increasing attention to virome complexity, studies of feline gastrointestinal disease have increasingly examined the intestinal bacteriome as a potential contributor to, or marker of, enteric disease outcomes [29,30,31,32]. In cats, acute diarrhea of diverse etiologies has been associated with reduced microbial diversity and expansion of facultative or opportunistic taxa [30,31]. However, direct FPV-specific characterization of the gut bacteriome remains limited, and current interpretation relies largely on feline enteritis or acute-diarrhea proxy data rather than FPV-confirmed longitudinal bacteriome cohorts [30,31,32]. The extent to which FPV-specific mechanisms, such as crypt necrosis, immune suppression, altered nutrient flux, and treatment exposure, shape microbial community structure and recovery trajectories remains incompletely defined.

The aim of this review is to synthesize current knowledge on viral enteritis in shelter cats using FPL as an anchor condition, with emphasis on viral co-detection, diagnostic interpretation, gut microbiome disruption, and recovery dynamics. The novelty of the review lies in reframing FPV-associated enteritis as a time-structured ecological perturbation rather than only as a classical viral disease. This integrative approach connects shelter exposure pressure, diagnostic ambiguity, enteric virome co-detection, proxy bacteriome evidence, treatment-related confounding, and recovery dynamics within a single evidence-bounded framework. It also distinguishes direct FPV evidence from syndrome-level feline proxy evidence, comparative non-feline data, and mechanistic hypotheses, thereby helping to define priorities for future longitudinal virome–bacteriome studies.

**Figure 2:**
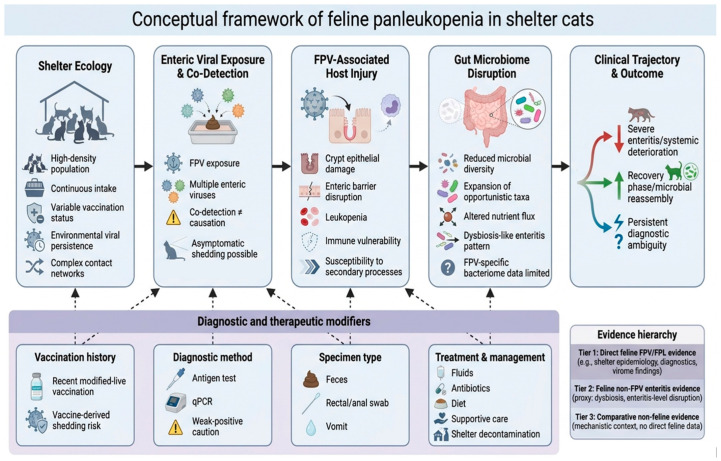
Conceptual framework of feline panleukopenia in shelter cats. The schematic shows the relationships among shelter ecology, viral exposure and co-detection, FPV-associated host injury, gut microbiome disruption, and clinical outcome. Diagnostic and therapeutic modifiers are shown below the main pathway, and the evidence hierarchy is summarized on the right. Colors and arrows are schematic.

The conceptual relationships among shelter ecology, viral exposure, host injury, microbiome disruption, and clinical outcomes, together with key diagnostic and therapeutic modifiers have been summarized in Figure 2.

This review is organized around three evidence-bounded propositions: first, FPV-associated disease in shelter cats occurs within a high-exposure enteric detection field in which co-detected viruses may represent exposure markers, secondary passengers, or potential disease modifiers [2,6,8,22,23]. Second, although direct FPV-associated bacteriome data remain limited, FPV enteritis provides a biologically plausible model for a time-structured gut ecosystem perturbation [30,31,32,33,34,35,36]. Third, progress in this field requires longitudinal, multi-layer study designs capable of separating direct disease mechanisms from shared shelter exposure, diagnostic confounding, and proxy-level inference [23,27,32,37]. These propositions are treated as testable hypotheses rather than established conclusions.

### Evidence Tiers and Boundaries of Inference

Because direct FPV-specific gut bacteriome data remain limited, this review uses an evidence-tiered framework to separate direct evidence from proxy evidence and mechanistic inference. Direct feline FPV/FPL evidence is considered Tier 1 and includes studies on FPV detection, clinical feline panleukopenia, shelter epidemiology, diagnostic interpretation, vaccine-associated shedding, and feline enteric virome findings in clinically characterized populations [1,2,6,7,8,20,21,22,23,24,25,26,27,28]. This tier provides the strongest basis for statements on transmission ecology, diagnostic confounding, viral co-detection, and the limits of causal interpretation in virome studies [8,22,23,24,25,26,27,28].

Tier 2 consists of feline non-FPV enteritis evidence, including studies of acute feline diarrhea and related gastrointestinal syndromes [16,30,31,32]. These studies are used as enteritis-level comparators because they involve clinically relevant intestinal disturbance, diarrhea-associated dysbiosis, reduced microbial diversity, and expansion of opportunistic taxa. However, they are not treated as evidence of FPV-specific microbial signatures, because most available studies do not combine confirmed FPV infection with longitudinal bacteriome profiling, clinical severity data, and recovery-phase sampling [30,31,32].

Tier 3 includes comparative, mechanistic, and conceptual evidence, including canine parvoviral enteritis, broader host–microbiome studies, and ecological host–microbiome–pathogen frameworks [17,29,38,39,40,41,42,43,44,45,46]. These sources are used to support mechanistic orientation and hypothesis generation regarding severe viral enteritis, colonization resistance, microbial ecosystem disturbance, recovery, reassembly, and host–microbiome dynamics [17,29,38,39,40,41,42,43,44,45,46]. They do not substitute for feline FPV-specific data and are not used to make definitive claims about FPV-associated microbiome structure, recovery trajectories, or intervention effects.

The tier assigned to each claim depends on study host species, disease specificity, diagnostic certainty, sampling design, temporal resolution, and whether the measured outcome directly addresses FPV, enteritis-associated dysbiosis, or a broader mechanistic model. This hierarchy is summarized in Table 1 and is used throughout the review to distinguish conclusions supported by direct evidence from cautious inference and hypothesis-generating interpretation.

## 2. Enteric Virome and Co-Detection Ecology in Shelter Cats

Shelter cats exist within enteric viral environments shaped by continuous intake, high contact density, and variable vaccination status [8,10]. In this context, vaccination histories are often heterogeneous because cats may arrive unvaccinated, incompletely vaccinated, vaccinated before shelter entry with incomplete records, or with unknown vaccination status [8,10]. Some cats may also have been recently vaccinated with modified-live vaccines, which can complicate FPV interpretation because vaccine-associated shedding may be detected by molecular or antigen-based assays [24,25,27]. These differences affect both susceptibility to FPV infection and interpretation of viral detection [23,24,25,26,27]. Consequently, the feline enteric virome in shelters is better conceptualized as an exposure landscape shaped by population turnover, shared environmental contamination, and frequent viral co-detection [7,8,10,20,21,22,23], rather than as direct evidence of causative pathogens [22,23]. Viral detection in this setting may reflect active infection, subclinical carriage, recent exposure, or vaccine-associated signal, depending on clinical and temporal context [20,21,22,23,24,25,27]. This interpretation shows that carnivore parvoviruses are ecologically flexible and capable of crossing species barriers [4].

At the molecular level, host-range shifts are influenced mainly by variation in the viral capsid protein VP2, particularly surface-loop residues that affect binding to the host transferrin receptor and antibody recognition [47,48]. Successful infection then depends on receptor-mediated entry, intracellular trafficking and uncoating, nuclear replication in rapidly dividing cells, amplification in target tissues, and fecal shedding, which together provide a mechanistic basis for ecological flexibility and interspecies transmission [3,4,47,48,49].

Metagenomic and targeted analyses consistently identify diverse viral communities in feline feces, including parvoviruses, astroviruses, caliciviruses, bocaparvoviruses, and other small DNA and RNA viruses [50,51,52,53,54,55]. These agents are detected in both clinically normal and diarrheic cats, limiting direct etiological attribution [21,56,57]. Co-detection should be regarded as an epidemiological signal rather than evidence of interaction or synergistic pathogenicity [20,21,22,23]. This distinction is reinforced by observations of genetically heterogeneous parvovirus populations and co-circulating parvovirus species in naturally infected cats [28]. In shelter conditions, shared exposure pressure can generate clusters of detections that resemble causal associations without demonstrating them [20,22,23].

Evidence for virome-level differences in feline panleukopenia derives primarily from case–control studies showing altered viral composition and differential co-virus detection frequencies in affected cats compared with controls [22]. However, these findings remain correlational and do not resolve temporality or causality [22,23]. Additional viral detections may precede disease, arise secondary to epithelial and immune disruption, or reflect shared environmental exposure [22,23]. Outbreak-based molecular analyses further indicate that FPV epizootics may involve distinct viral lineages while maintaining relatively limited intra- and interhost diversity within outbreaks [58,59]. Shelter studies also show that fecal FPV DNA shedding can persist beyond the acute clinical phase, whereas clinical signs and antigen results do not reliably track ongoing shedding [60]. Together, these observations support a distinction between outbreak phylogenetic structure, viral co-detection, and host-level mechanisms [22,23,58,59,60].

Methodological and biological factors further complicate interpretation. Virome profiles vary with sampling time, sequencing depth, enrichment protocols, and analytic thresholds, while diarrhea can alter relative abundance through dilution and transit effects [23,45]. A substantial proportion of detected viral sequences remains unclassified, limiting precise attribution [45,50]. Bacteriophages may indirectly influence bacterial community structure, meaning that virome signals may reflect broader microbial ecosystem shifts rather than direct eukaryotic infection [45]. Commensal viral populations may also contribute to mucosal immune regulation, challenging strictly pathogen-centered interpretations of enteric virome data [61].

Diagnostic interpretation introduces an additional constraint, and molecular methods are important for interpreting FPV detection in shelter cats, particularly in studies of enteric viromes, gut microbiome disruption, and dysbiosis [22,23,27,30,32]. Without strain-discriminating, quantitative, or temporal interpretation, FPV DNA detection may be misclassified as active infection when it may instead reflect recent modified-live vaccination, residual viral DNA, prolonged shedding, subclinical carriage, or environmental contamination [23,24,25,26,27,60]. Early PCR assays established the basis for molecular detection of carnivore parvoviruses [62], while later approaches such as PCR-RFLP, real-time PCR, minor-groove-binder probe assays, VP2 sequencing and high-resolution melting analysis improved sensitivity, FPV/CPV differentiation, molecular epidemiological resolution, and discrimination between vaccine-derived and field-virus signals [63,64,65,66,67,68]. These methods are particularly useful in shelter outbreaks, low-antigen samples, atypical clinical presentations, and retrospective analyses of fecal, rectal, blood, tissue, or environmental samples [8,23,27,60]. However, molecular positivity alone does not confirm the presence of infectious virus or causative disease [23,27,60]. Diagnostic interpretation should therefore integrate assay characteristics, vaccination history, timing of sampling, clinical signs, leukocyte counts, antigen test results, viral load when available, and epidemiological context [23,24,25,26,27,60]. This distinction is also important for microbiome and virome studies, where vaccine-derived or residual DNA signals may inflate apparent prevalence and complicate interpretation of microbial community shifts [23,24,25,26,27,60]. Because sequencing or strain-discriminating assays are not yet routinely implemented in many shelter settings [63,65,67,68,69], FPV detection should be interpreted within a structured framework that separates exposure, infection, disease, and secondary microbial disruption (Table 2).

Current evidence therefore supports a restrained interpretation: the shelter enteric virome provides an important ecological context for FPV-associated disease, but it does not provide a direct map of causative interactions [20,21,22,23]. Future studies should combine longitudinal virome and bacteriome sampling with validated diagnostic classification, vaccination metadata, viral load information, and clinical severity metrics [23,27,32,37,60]. Such designs are needed to determine whether co-detected viruses function as exposure markers, opportunistic passengers, or true modifiers of disease trajectory [22,23,60].

Evidence status. The strongest evidentiary support in this section derives from direct studies of feline shelter populations, including virome analyses, molecular epidemiological studies, shelter outbreak investigations, and diagnostic investigations in feline panleukopenia [8,20,21,22,23,27,58,59,60] (Tier 1). In contrast, interpretation of broader virome architecture, particularly phage–bacteria interactions and the contribution of unclassified viral sequence space, draws partially on general virome ecology literature [45,50,61] (Tier 3). These elements therefore provide mechanistic context rather than FPV-specific evidence. The present section supports a restricted inference: shelter environments function as high-exposure systems [7,8,10] in which viral co-detection is common [20,21,22], but current data do not establish causal or synergistic interactions among co-detected viruses [22,23].

## 3. FPV Enteritis as a Time-Structured Perturbation of the Gut Ecosystem

Feline panleukopenia provides a biologically plausible model for gut ecosystem disruption because its pathogenesis generates a sharply time-structured intestinal injury with downstream consequences for microbial community stability [1,2,6,13,14]. FPV replicates preferentially in rapidly dividing cells, especially intestinal crypt epithelial cells, lymphoid tissues, and bone marrow [1,2,6,13,14]. In the intestine, FPV targets mitotically active crypt epithelial cells, including the stem/progenitor cell compartment responsible for continuous epithelial renewal. Loss of these proliferating crypt cells reduces replacement of mature villous enterocytes, so damaged or senescent epithelial cells are not adequately renewed. As a result, crypt infection leads to crypt necrosis, villous blunting or collapse, mucosal barrier disruption, and altered intestinal absorption [1,2,6,13,14]. These changes may modify the luminal environment by increasing inflammatory exudation, changing nutrient and substrate availability, altering oxygen gradients, and disrupting normal mucosa–lumen exchange [38,39,70]. At the same time, lymphoid depletion and bone marrow suppression contribute to leukopenia and reduced immune containment, which may further favor opportunistic microbial expansion [1,2,6,13,14]. These downstream consequences could weaken colonization resistance and reshape microbial competition within the disturbed gut ecosystem, although they have not yet been directly demonstrated in naturally infected cats with FPV [39,41].

From a microbiome perspective, FPL-associated enteritis is best approached as a sequence of disease and recovery phases rather than as a single diagnostic state. During acute disease, epithelial injury, inflammation, leukopenia, and altered intestinal transit may reshape microbial competition and resource use [1,2,6,13,14,38,39,40,70]. In the absence of FPV-specific longitudinal bacteriome data, studies of feline acute diarrhea provide the closest syndrome-level reference, consistently reporting reduced microbial diversity and increased representation of facultative or opportunistic taxa [30,31,32]. These findings should be treated as enteritis-associated patterns and not assumed to represent FPV-specific signatures without direct confirmation [30,31,32].

The early recovery period may be particularly informative, as microbial communities reassemble under conditions of incomplete barrier restoration. During this phase, outcomes may diverge, including recovery toward a prior state or stabilization in an altered configuration [32,40,46]. Clinical management can influence these trajectories. Antibiotic therapy, often justified in severe FPV because of the risk of bacterial translocation in the context of mucosal injury and leukopenia, may simultaneously reduce microbial diversity and constrain recolonization pathways [33,34,35,36]. Therapeutic interventions should be considered active ecological modifiers rather than neutral background variables [33,34,35,36].

A phase-based framework also clarifies the types of endpoints required for mechanistic interpretation. Taxonomic composition alone is unlikely to capture functional consequences of microbiome disruption. Metabolic outputs, including short-chain fatty acids, bile acids, and tryptophan-derived compounds, provide more direct links to host barrier function and immune signaling. Evidence from feline acute diarrhea indicates that compositional changes may coincide with substantial metabolite shifts, supporting the integration of metabolomic and microbial data [34,36]. For FPV, informative designs would include repeated sampling across disease onset and recovery, combined with clinical indicators such as leukopenia severity, leukocyte recovery, duration of hospitalization, and survival, to distinguish functional recovery from clinical improvement [6,65,66,67].

This hypothesis-generating framework supports three priorities: definition of appropriate baselines, including age and environmental context, systematic recording of therapeutic and dietary exposures and longitudinal sampling rather than single-time-point analysis [32,37,71,72,73,74,75,76,77,78]. Under these conditions, FPV can be investigated as a temporally evolving perturbation of the gut ecosystem, with effects that may differ across phases of disease and recovery [32,40,46].

**Figure 3:**
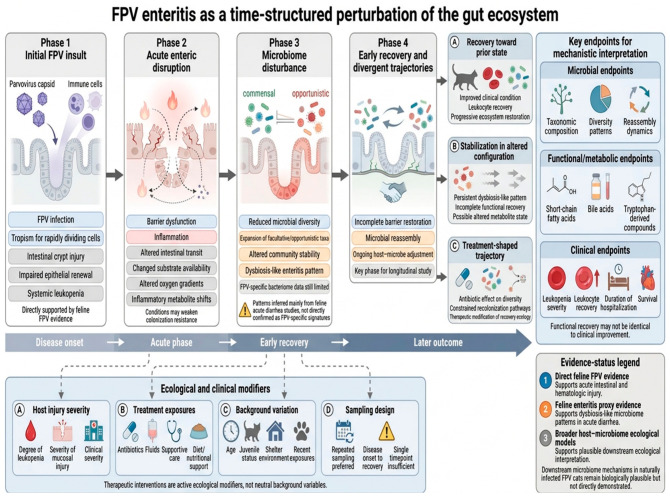
FPV enteritis as a time-structured perturbation of the gut ecosystem. The schematic shows FPV enteritis as a temporal process from initial viral injury to acute enteric disruption, microbiome disturbance, early recovery, and possible later outcomes. Solid arrows indicate disease progression, dashed arrows indicate modifying factors, and colors are used schematically to distinguish clinical, microbial, functional, and evidence-level elements.

Because these effects are phase-dependent, interpretation of microbiome data requires careful separation of disease-related changes from background variation associated with age, environment, and recent exposures. The following section therefore addresses baseline definition in feline populations, with emphasis on shelter and juvenile contexts (Figure 3).

Evidence status. Direct feline FPV evidence in this section primarily supports the acute intestinal and hematologic injury associated with disease [1,2,6,13,14] (Tier 1). Downstream ecological interpretations, including effects on colonization resistance, resource structure, and microbiome stability, are inferred from feline enteritis proxy data [30,31,32] (Tier 2) and broader host–microbiome ecological models [38,39,40,41,43,46] (Tier 3). These interpretations should therefore be regarded as biologically plausible, conceptual, and hypothesis-generating extensions rather than directly demonstrated FPV-specific mechanisms.

## 4. Defining Reference Baselines for Microbiome Interpretation in Household, Shelter, and Juvenile Cats

Interpretation of microbiome data in feline panleukopenia depends on the definition of an appropriate reference baseline, meaning the comparison state against which disease-associated microbial changes are evaluated [32,71,72,73,74]. A single universal “normal” feline microbiome is unlikely to be suitable because clinically healthy cats may differ substantially according to age, housing environment, diet, medication exposure, stress, and recent pathogen exposure [71,72,73,74,76,77,78]. In shelter medicine, this variability is intrinsic to the system and should be modeled rather than ignored [6,32,71,74].

Environmental context is a primary determinant of baseline configuration [79,80,81,82]. Household cats generally experience more stable diets, lower pathogen exposure, and more consistent medical care [71,72,73,74], whereas shelter cats are exposed to dense contact networks, frequent intake of new animals, dietary changes, and stress-associated physiological responses [6,10,37,71]. These conditions may shape microbial communities independently of overt disease [32,71,72,73,74]. Therefore, household controls cannot be assumed to represent an appropriate comparator for shelter-associated FPV without adjustment for environmental effects [32,71,72,73,74]. Comparative data from canine parvoviral dysbiosis may inform general mechanisms [42], but do not substitute for feline-specific baseline definition [30,31,32,71,72,73,74].

Age is another major source of baseline variability. FPV predominantly affects juveniles, whose microbiome is still developing and may be more responsive to perturbation than that of adults [6,75,76]. Early-life microbial communities are influenced by dietary transitions, immune maturation, and environmental exposure [75,76], resulting in developmental trajectories that differ across individuals [71,75,76]. Comparisons between affected juveniles and adult controls may therefore conflate developmental dynamics with disease-associated change [71,75,76]. Age-matched cohorts are needed to separate age-related microbiome variation from FPV-associated microbial changes [6,71,75,76].

Dietary, pharmacological, and temporal factors also complicate baseline interpretation. Abrupt diet changes, antibiotics, and other medications can alter microbial composition and metabolic output [34,35,36,77,78,79]. Because these exposures often coincide with shelter entry and FPV treatment, they should be treated as part of the ecological context rather than as secondary confounders [2,6,32,33,34,35,36,79]. Temporal factors introduce an additional constraint because single-time-point microbiome comparisons cannot distinguish pre-existing baseline variation from disease-associated change or recovery-phase effects [32,75,76,80]. Longitudinal sampling from intake through recovery provides a stronger basis for reconstructing within-animal trajectories [32,75,76,80].

These considerations support a layered baseline framework rather than a single universal reference state. At minimum, FPV microbiome studies should include shelter-matched clinically unaffected cats to capture environmental background [6,32,71,74], age-matched cohorts to control for developmental effects [71,75,76], and longitudinal sampling to capture dynamic change [32,75,76,80]. In practice, these reference layers can be implemented using standardized metadata collection, matched control selection, repeated fecal sampling, stratification by age or housing status, and statistical approaches adjusted for diet, treatment, medication exposure, and shelter duration [32,37,71,74,80]. Using these layers together may help distinguish disease-associated signals from environmental and developmental variability.

Evidence status. The importance of context-matched baselines in cats is supported by feline microbiome studies and population-context analyses [71,72,73,74,81,82,83]. However, the specific baseline structure required for FPV microbiome interpretation in shelter juveniles remains incompletely defined because direct FPV-specific bacteriome datasets are limited [30,31,32]. The framework proposed here should therefore be regarded as a methodological extension grounded in available evidence rather than an established standard validated in FPV-specific cohorts [32,71,74].

## 5. Acute Enteric Perturbation and Dysbiosis: Proxy Evidence and Constraints

In the absence of FPV-specific longitudinal bacteriome datasets, interpretation of microbial dynamics in feline panleukopenia must draw on syndrome-level evidence from studies of acute feline diarrhea and broader enteric microbiome disturbance [30,31,32]. This extrapolation is biologically plausible because FPV induces epithelial injury, disrupts nutrient flux, and activates inflammatory and immune-mediated responses that may reshape intestinal microbial communities [1,2,6,29,38,39]. Evidence from enteric viral infections more broadly supports interactions between viral injury, barrier integrity, and microbiome structure, although direct FPV-specific bacteriome data remain limited [29]. Under these conditions, patterns observed in diarrhea cohorts should be treated as indicators of severe enteritis rather than as FPV-specific microbial signatures unless confirmed in targeted studies [30,31,32].

Across feline acute diarrhea datasets, a recurring but non-specific profile has been reported, including reduced alpha diversity, depletion of obligate anaerobes, and relative expansion of facultative or opportunistic taxa, including members of *Enterobacteriaceae* and other aerotolerant groups [30,31,32]. These changes match disturbed gut environments characterized by altered oxygen tension, modified nutrient availability, impaired host defenses, and reduced colonization resistance [38,39,41,43,70]. This pattern is best interpreted as a community-level disturbance rather than as the emergence of a single dominant pathogen, supporting the interpretation of dysbiosis as a system-level shift [16,39,41].

Direct extrapolation to FPV remains constrained by several factors. Acute diarrhea cohorts are etiologically heterogeneous, combining infectious, dietary, and idiopathic causes that may produce overlapping but non-identical microbial responses [30,31]. Sampling is often cross-sectional and may occur at variable disease stages, limiting temporal resolution [32,80]. In addition, treatment effects, especially those related to antibiotics, fluid therapy, and dietary changes, are often difficult to separate from disease progression [2,6,32,33,34,35,36,77,78,79]. These constraints define the limits of proxy-based inference. Comparable work in canine enteropathy shows that dysbiosis can be quantified and structured without being disease-specific, reinforcing the distinction between detecting ecological disturbance and assigning disease-specific meaning [84,85].

FPV-associated dysbiosis is therefore best viewed as a temporally evolving response to acute intestinal injury, but this view remains hypothesis-generating until FPV-specific longitudinal bacteriome data are available [1,2,6,30,31,32]. During the acute phase, microbial disruption may be most pronounced, with reduced diversity and expansion of opportunistic taxa reflecting barrier compromise, inflammation, altered transit, and modified resource conditions [30,31,32,38,39,41,70]. Early recovery represents a transitional phase in which microbial communities may reassemble under continued selective pressures, including ongoing treatment and diet change [33,34,35,36,77,78,79]. Later stages may involve returning toward a prior configuration or stabilization in an altered state, depending on host factors, environment, and therapeutic exposure [32,40,46,80].

Taxonomic composition alone provides limited insight into functional recovery. Evidence from feline and comparative systems indicates that similar microbial profiles can correspond to different metabolic outputs, while functional redundancy may mask underlying instability [30,32,40,86,87,88]. For this reason, assessment of dysbiosis should extend beyond composition to include functional measures such as short-chain fatty acids, bile acids, and other microbiome-derived metabolites that more directly reflect host–microbe interactions [30,32,89,90].

Interpretation also requires restraint. Reduced diversity or expansion of opportunistic taxa in FPV would not establish a causal role in disease severity and may arise secondary to epithelial damage, immune suppression, treatment exposure, or shelter-associated environmental pressures [1,2,6,30,31,32,33,34,35,36]. Similar microbiome alterations are reported across a range of feline gastrointestinal conditions, and comparable findings in canine enteropathy indicate that dysbiosis patterns are not disease-specific [84,85,91]. Establishing FPV-specific relevance would require longitudinal designs linking microbial configurations to clinical outcomes such as leukopenia severity, hospitalization duration, and survival [6,32,92,93].

Current evidence supports a limited conclusion: FPV-associated enteritis may be accompanied by dysbiosis consistent with acute intestinal disturbance, but its composition, functional impact, and clinical relevance remain insufficiently defined in FPV-specific cohorts [30,31,32]. Until such data are available, microbiome changes should be interpreted as structured responses to enteritis rather than defining features of FPV pathogenesis. Related feline viral-disease studies further support the importance of time-resolved analysis of microbiome change during infection and treatment [94].

Progress in this area will depend on integrated study designs combining bacteriome profiling with virome data, clinical parameters, and explicit temporal sampling [32,37,80,86,87]. Such approaches would help distinguish the relative contributions of viral injury, secondary microbial shifts, and therapeutic interventions in FPV-associated disease. The current evidence can therefore be organized as a combined virome–bacteriome framework that differentiates direct feline data from proxy evidence and mechanistic inference (Table 3).

Evidence status. This section relies primarily on feline acute-diarrhea studies as syndrome-level proxy evidence [30,31,32] (Tier 2), rather than direct FPV-associated bacteriome data. Mechanistic interpretation is further informed by broader work on disturbed gut ecosystems, colonization resistance, and microbiome function [38,39,40,41,43,46,70] (Tier 3). Accordingly, reduced diversity and expansion of opportunistic taxa should be interpreted as general enteritis-associated responses, not as confirmed FPV-specific microbial signatures.

## 6. Recovery, Reassembly and Ecological Trajectories

Recovery from feline panleukopenia should be approached as a progressive process rather than as a discrete transition from disease to health, with potential reorganization of the intestinal ecosystem after acute injury [1,6,32]. In shelter populations, clinical outcome is heterogeneous and influenced by measurable prognostic factors, including leukopenia severity and supportive care intensity [93]. Importantly, resolution of clinical signs, such as cessation of vomiting or return of appetite, does not necessarily indicate restoration of microbial community structure or function. Clinical recovery, microbial composition, and metabolic activity may evolve on different timescales [32,40,90].

As a conceptual and hypothesis-generating framework, three broad recovery trajectories can be proposed: return toward a pre-perturbation state, stabilization in an alternative configuration, or persistent instability [40,46,95,96]. Persistent instability would involve fluctuating community structure and incomplete recovery of ecological functions, potentially reducing resilience to subsequent stressors [32,90]. These trajectories should be interpreted as heuristic models rather than empirically validated recovery classes in FPV.

Trajectory divergence may be shaped by interacting host, environmental, and treatment-related factors. Host characteristics, including age, immune competence, and depth of leukopenia, may interact with shelter-specific conditions such as exposure pressure and diet variability [6,76,77]. Interventions applied during acute disease may further shape recovery. Antibiotic therapy, while often necessary because of the risk of bacterial translocation in the context of mucosal injury and leukopenia, can reduce microbial diversity and alter community structure, potentially influencing subsequent reassembly [33,34,35,36]. Evidence in cats shows that antimicrobial exposure alone can induce measurable metabolic changes, supporting a direct ecological effect of treatment [79]. Nutritional support and gradual dietary stabilization may also influence recovery by restoring substrate availability for commensal taxa [40,76,77,78]. The early post-acute phase may therefore represent a period of increased sensitivity, during which microbial communities are reassembling and ecological niches may remain incompletely occupied [32,90].

A major limitation in current FPV research is the absence of longitudinal datasets capable of tracking these processes over time. Most available data are cross-sectional or limited to short-term clinical observation, preventing distinction between stable recovery and transient improvement [32,80]. Evidence from other feline viral conditions indicates that gut microbial communities can shift dynamically during infection and treatment, supporting the need for time-resolved sampling [94]. However, it remains uncertain whether normalization of clinical signs corresponds to restoration of microbial function or whether subclinical instability persists after discharge. Comparative parvoviral studies suggest that post-acute effects may extend beyond the initial disease phase, although these findings cannot substitute for feline-specific FPV microbiome data [44]. Addressing this gap requires sampling strategies that extend from intake through convalescence and incorporate both compositional and functional measures [32,80,90].

Functional recovery warrants particular attention. Changes in taxonomic composition do not necessarily indicate restoration of key metabolic processes [32,86,87]. Recovery of functions such as short-chain fatty acid production, bile acid metabolism, and host–microbe signaling may lag behind compositional shifts or follow different trajectories [30,89,90]. For this reason, assessment of recovery should include functional endpoints in addition to taxonomic profiling [30,32,86,87,90].

In shelter environments, recovery occurs under continued infectious and environmental pressure. Unlike household settings, where post-recovery conditions may be relatively stable, shelter cats remain within high-density contact networks characterized by ongoing intake, exposure, and environmental contamination [2,6,8,10,37]. Ongoing exposure could limit ecological recovery or favor persistence of altered community states or recurrent disturbance, although this remains to be confirmed in FPV-specific longitudinal studies [32,71]. Taken together, FPV-associated recovery is best approached as a temporally evolving process shaped by host condition, environmental context, and clinical management [6,32,90].

Until longitudinal, multi-layer datasets are available, clinical resolution should not be assumed to reflect complete ecological recovery. This perspective provides a basis for evaluating interventions that may influence recovery dynamics. The following section therefore examines therapeutic and supportive strategies in FPV.

Evidence status. The recovery trajectories described here are conceptual and hypothesis-generating. Direct FPV evidence supports heterogeneous clinical recovery and the prognostic relevance of factors such as leukopenia and supportive care [6,93] (Tier 1), but it does not yet establish defined microbiome recovery trajectories. The categories of return, alternative stabilization, and persistent instability are proposed as heuristic models informed by ecological theory, comparative microbiome research, and limited feline data [32,40,44,46,94,95,96] (Tier 3), rather than as empirically established classes in FPV.

## 7. Interventions as Ecological Modifiers in FPV-Associated Enteritis

The interventions discussed below are not presented as validated microbiome-restorative therapies in feline panleukopenia, but as factors that may influence microbial reassembly during recovery. Their effects are biologically plausible yet remain insufficiently characterized in FPV-specific contexts.

In clinical practice, therapeutic and supportive measures in feline panleukopenia are primarily evaluated through outcomes such as survival, resolution of clinical signs, and duration of hospitalization [1,2,6,93]. From a microbiome perspective, however, these interventions may also act on a disrupted intestinal ecosystem. Rather than restoring a predefined “normal” state, they may introduce selective pressures that alter the direction and stability of microbial recovery, with effects depending on timing, intensity, host condition, and environmental context [32,33,34,35,36,97].

Antibiotic therapy is one of the most consequential interventions in severe FPV-associated enteritis. Its use may be clinically justified by the risk of bacterial translocation associated with mucosal injury, leukopenia, and impaired host defense [1,2,6,14]. At the same time, antibiotics are known to reduce microbial diversity and alter community composition, with effects that may persist beyond the treatment period [34,35,36]. In FPV, this creates a practical tension between mitigation of systemic infection risk and potential disruption of microbial recovery. Antibiotic exposure during acute disease may influence the initial conditions under which microbial communities reassemble, although the magnitude and duration of these effects in FPV remain unclear and are largely inferred from broader microbiome studies [34,35,36,79].

Nutritional management represents a second major influence. Diet determines substrate availability for microbial metabolism and therefore shapes both community composition and functional output [76,77,78]. In shelter settings, dietary changes frequently occur at intake and may continue throughout illness and recovery, introducing variability that is rarely standardized [6,10,76,77]. Gradual refeeding and dietary stabilization may support microbial recovery by restoring consistent substrate supply, including substrates relevant to short-chain fatty acid production, but direct evidence linking specific feeding strategies to microbiome or clinical outcomes in FPV is limited [40,77,78].

Probiotics are widely used in companion animal medicine, but their role in FPV remains uncertain [98,99]. Their effects depend on the strain used, timing of administration, host condition, and interactions with the resident microbial community [98,99]. In acute enteritis, where colonization resistance may be impaired and the microbial ecosystem unstable, introduced organisms may have transient or variable effects [39,41,98,99]. Some shelter-based studies in mixed populations of cats and dogs report modest reductions in diarrhea duration, but these findings are not specific to FPV and should therefore be interpreted cautiously [100]. Claims that probiotics restore the FPV-associated microbiome should be avoided until supported by controlled FPV-specific studies.

Two general considerations are relevant across interventions. First, timing influences impact: treatments applied during acute epithelial injury, early recovery, or later stabilization act on different system states and may therefore produce different outcomes [32,90]. Second, intervention effects must be interpreted within the shelter context, where exposure pressure, variable nutrition, population turnover, and environmental contamination may limit the applicability of data derived from more controlled settings [6,10,32,37,71].

Available evidence supports a cautious interpretation. Interventions in FPV-associated enteritis may influence the course of microbial recovery, but current data do not show that they reliably direct recovery toward a defined microbiome endpoint [32,90,97]. Their effects depend on host condition, environmental context, and timing, and remain insufficiently characterized in FPV-specific studies. For this reason, claims regarding microbiome-targeted therapeutic benefit should be reserved until supported by longitudinal and controlled investigations integrating microbial, functional, and clinical outcomes.

This perspective supports a research approach in which interventions are evaluated not only by clinical endpoints but also by their effects on microbial reassembly. The following section therefore outlines key methodological priorities for advancing the study of gut ecosystem dynamics in feline panleukopenia.

Evidence status. Clinical justification for supportive treatment and antimicrobial use in severe FPV is grounded in feline disease management literature [1,2,6,14,93] (Tier 1), whereas claims about microbiome-directed consequences and trajectory modification rely largely on broader microbiome and comparative intervention literature [33,34,35,36,79,97,98,99,100] (Tier 3, with limited Tier 2 support). Therefore, the intervention model proposed here should be interpreted as a hypothesis-generating ecological framework rather than evidence that specific treatments restore the FPV-associated microbiome.

## 8. Research Agenda, Methodological Priorities and Limitations

The current evidence base for feline panleukopenia in shelter environments supports an ecological interpretation, but remains limited by cross-sectional designs, reliance on proxy microbiome data, and incomplete integration of virome, bacteriome, diagnostic, and host-level information [22,23,32,82]. Progress therefore requires longitudinal, multi-layer cohort studies in shelter populations, with sampling across intake, acute disease, early recovery, and late convalescence [6,32,37,80]. Shelter-specific variability, including incomplete background histories, heterogeneous intake pathways, vaccination status, age structure, diet, and management context, should be incorporated as structured study variables rather than treated as incidental variation [6,10,32,37]. Standardized metadata collection is therefore essential for improving comparability and reducing diagnostic ambiguity (Table 4).

Longitudinal frameworks offer a practical advantage because each animal can serve, in part, as its own reference over time. Sampling should explicitly include intake, post-vaccination intervals, onset of clinical signs, early recovery, and late convalescence [24,25,32,60,80]. Without this temporal resolution, it is difficult to determine whether observed microbiome or virome changes precede disease, arise during clinical decline, reflect vaccination or treatment effects, or persist after apparent recovery [22,23,27,32,60].

A second priority is integration of multiple analytical layers. Future studies should combine bacteriome profiling, such as 16S rRNA sequencing or shotgun metagenomics, with virome characterization and metabolomic assessment of functionally relevant outputs, including short-chain fatty acids, bile acids, and tryptophan-derived metabolites [30,32,40,82,86,87]. These data should be interpreted alongside clinical endpoints, including survival, disease duration, relapse, leukopenia depth, leukocyte recovery kinetics, hospitalization duration, and systemic complications [6,13,92,93]. Functional measures, metabolite profiles, and markers of barrier integrity are especially important for linking microbial patterns to host physiology [30,32,88,89,90].

Diagnostic refinement remains necessary for reducing misclassification. Future studies should record vaccination timing relative to sampling and, where possible, include quantitative molecular outputs, such as Ct values, together with strain-discriminating methods that help distinguish vaccine-derived signal from field infection [23,24,25,26,27,60,63,101]. Ongoing molecular characterization of FPV strains may also clarify the genetic context of outbreaks and diagnostic findings [58,59,63,102].

Contextual documentation is equally important. At minimum, studies should report age, housing conditions, vaccination status and timing, diet and dietary transitions, antimicrobial and supportive therapies, retroviral status where available, and clinical severity indicators [6,32,37,71,76,77,78,103]. Without structured metadata, comparisons across studies remain unreliable and conclusions difficult to generalize [32,37,71,82].

Where FPV-specific data are limited, comparative insights from other species or enteric conditions may inform hypothesis generation, but should not be treated as substitutes for feline FPV evidence [32,40,44,90]. Likewise, pathogen detection in shelter systems must be interpreted within exposure and diagnostic constraints, rather than as direct evidence of interaction or causation [20,21,22,23,27]. Future work should therefore prioritize temporal sequence, functional impact, and interaction structure over presence–absence comparisons alone [32,37,80,86,87].

### Limitations of the Current Evidence Base

Interpretation of the current literature is constrained by several recurring limitations. First, most virome and microbiome data in shelter cats are cross-sectional, limiting assessment of temporal sequence, causal direction, persistence, and recovery [20,21,22,23,30,31,32]. Second, diagnostic uncertainty, especially vaccine-associated shedding and incomplete vaccination metadata, can lead to misclassification of FPV positivity [23,24,25,26,27,60]. Third, sampling and disease-stage effects, including diarrhea-associated dilution and altered intestinal transit, may influence both virome and bacteriome profiles [23,30,31,32,45]. Fourth, clinical management, including antibiotics, fluid therapy, dietary modification, and supportive care, often coincides with disease progression and may independently shape microbial communities [2,6,32,33,34,35,36,77,78,79]. Finally, baseline definition remains difficult in juvenile shelter cats because age, pre-intake exposure, stress, diet, and subclinical disease can all contribute to microbiome variability [32,71,75,76]. Comparative evidence from canine parvoviral enteritis or broader host–microbiome models can inform hypothesis generation, but cannot substitute for direct feline FPV-specific data [42,44,90].

These priorities define a research agenda centered on longitudinal, integrated, and context-controlled investigation of FPV-associated enteritis [32,37,80]. The key unresolved issue is not only whether microbiome and virome changes occur, but whether they influence disease trajectory, recovery, and long-term resilience. Addressing this question requires temporally resolved, multi-layer datasets and careful integration of ecological and clinical perspectives [32,37,40,80,86,87]. Until such evidence is available, interpretations should remain bounded and explicitly linked to the limits of the current data [22,23,30,31,32].

Evidence status. The priorities outlined in this research agenda reflect the imbalance between relatively strong descriptive evidence in feline FPV epidemiology, diagnostics, and shelter virome studies [8,20,21,22,23,24,25,26,27,60] (Tier 1) and limited direct evidence for FPV-associated gut ecosystem dynamics [30,31,32] (Tier 2/proxy evidence). Recommendations such as longitudinal sampling, integrated multi-omics, and function-oriented recovery assessment are therefore methodological responses to these gaps [32,37,40,80,86,87], rather than conclusions supported by an established FPV-specific microbiome evidence base.

## 9. Conclusions

Feline panleukopenia in shelter cats should be interpreted not only as an individual viral infection, but also as an ecological process shaped by host susceptibility, shelter exposure, diagnostic complexity, viral co-detection, and microbial community disturbance. Current evidence supports a cautious, hypothesis-generating framework in which virome co-detection and dysbiosis-associated patterns are not treated as direct proof of causation. Future longitudinal, context-controlled, and multi-layer studies integrating validated FPV diagnostics, virome and bacteriome profiling, clinical metadata, treatment records, and functional endpoints are needed to clarify the biological and clinical significance of gut ecosystem disruption in feline panleukopenia.

## Figures and Tables

**Table 1:** Evidence tiers and corresponding limits of inference in studies of feline panleukopenia virus (FPV), enteric viromes and microbiome disruption in shelter cats.

Evidence Tier	Type of Evidence	Role in This Review	Limit of Inference
Tier 1	Direct FPV/FPL evidence in cats	Supports statements on FPV pathogenesis, diagnosis, shedding, shelter outbreaks, prognosis, and virome findings.	Strongest relevance, but microbiome-specific data remain limited.
Tier 2	Feline enteric microbiome evidence not specific to FPV	Supports interpretation of diarrhea-associated dysbiosis, baseline variability, and feline gut ecosystem disturbance.	Indicates feline enteric patterns, but does not prove FPV-specific causation.
Tier 3	Comparative, mechanistic, or conceptual microbiome evidence	Provides context for dysbiosis, colonization resistance, recovery, reassembly, resilience, and functional disruption.	Hypothesis-generating only; cannot replace FPV-specific feline evidence.

**Table 2:** Key considerations for interpreting feline panleukopenia virus (FPV) detection in shelter cats.

Diagnostic Context	Possible Interpretation	What It Does Not Prove	Key Requirement
Antigen or PCR positive in a clinically ill cat	Possible active FPV infection	FPV as the only cause of disease	Integrate clinical signs, leukopenia, co-detection, and outbreak context
Strong qPCR positivity in a compatible, unvaccinated cat	High suspicion of active FPV infection	Disease severity or prognosis alone	Consider viral load, leukocyte count, and timing
Positive result after recent modified-live vaccination	Possible vaccine-associated detection or shedding	Field infection or clinical disease	Record vaccine type and timing
Low-level PCR positivity or late detection	Residual viral DNA, prolonged shedding, or contamination	Ongoing active disease	Assess viral load, recovery stage, and repeated tests
Viral detection in a clinically healthy cat	Exposure, subclinical infection, or asymptomatic shedding	Disease presence or progression	Consider shelter exposure and population turnover
Single-time-point detection	Viral material present at sampling	Temporal sequence or causality	Interpret with disease stage and sampling phase
Sequencing, PCR-RFLP, MGB probes, or HRM	Better strain-level interpretation	Routine availability or pathogenic proof	Use with clinical correlation
Viral co-detection in panels or metagenomics	Shared exposure or possible disease modifier	Synergistic pathogenicity	Consider co-detection bias, contamination, and microbiome disruption

**Table 3:** Interpretation of enteric virome and bacteriome dynamics in feline panleukopenia virus (FPV)-associated enteritis in shelter cats.

Domain	Pattern	What Current Evidence Supports	FPV Specific?	Main Limitation
Virome	Viral co-detection in shelter cats	FPV cases may show more Frequent co-detection of additional enteric viruses than controls	Partly	Cross-sectional data do not establish temporal order, interaction, or severity effects
Virome	Asymptomatic shedding and vaccine-associated signal	Detection may reflect exposure, carriage, or recent vaccination rather than active etiologic disease	Yes	Detection alone does not establish causation
Bacteriome	Reduced diversity and dysbiosis in feline acute diarrhea	Acute enteritis is associated with community disruption and loss of ecological stability	No	Enteritis-level proxy evidence, not FPV-specific profiling
Bacteriome	Expansion of facultative/ opportunistic taxa	Disturbed gut environments may favor aerotolerant or opportunistic groups	No	Cause–effect relationship remains unresolved
Functional layer	Metabolic disruption and loss of anaerobic function	Dysbiosis may affect SCFA production, bile acid metabolism and barrier-associated functions	No	FPV-specific functional data are lacking
Recovery layer	Incomplete or unstable ecological recovery	Clinical recovery may not coincide with microbiological or virological resolution	No	FPV-specific longitudinal trajectories remain insufficiently defined

**Table 4:** Core metadata domains required for interpretable studies of feline panleukopenia virus (FPV), enteric viromes and gut ecosystem disruption in shelter cats.

Domain	Minimum Variables	Why Required	Risk If Missing
Host background	Age, juvenile/adult status, retroviral status where available	Shapes susceptibility, severity and microbiome baseline	Confounding of disease and baseline effects
Vaccination context	Vaccine history and interval from vaccination to sampling	Needed to distinguish vaccine-associated detection from field infection	Misclassification of infection status
Clinical Phenotype	Leukopenia, gastrointestinal signs, dehydration, outcome	Links virome/ microbiome findings to biological relevance	Data remain descriptive only
Temporal Sampling	Sampling phase: intake, acute illness, recovery	Required to interpret detection, shedding and recovery dynamics	No temporal inference
Treatment Exposure	Antibiotics and major supportive therapies	Major modifiers of microbial trajectories	Disease effects cannot be separated from treatment effects
Shelter context	Density, turnover, cohorting/isolation practices	Defines exposure pressure and epidemiological background	Poor cross-study comparability
Diagnostic Context	Assay type, specimen type and key interpretation variables	Determines how positive results should be interpreted	Ambiguous diagnostic meaning

## Data Availability

The original contributions presented in this study are included in the article. Further inquiries can be directed to the corresponding author.

## Abbreviations

The following abbreviations are used in this manuscript:

ABCD	European Advisory Board on Cat Diseases
CPV	Canine parvovirus
Ct	Cycle threshold
DNA	Deoxyribonucleic acid
FCoV	Feline coronavirus
FIPV	Feline infectious peritonitis virus
FPL	Feline panleukopenia
FPV	Feline panleukopenia virus
HRM	High-resolution melting analysis
MGB	Minor-groove-binder
MLV	Modified-live vaccine
PCR	Polymerase chain reaction
PCR-RFLP	Polymerase chain reaction–restriction fragment length polymorphism
qPCR	Quantitative polymerase chain reaction
qPCR-HRM	Quantitative polymerase chain reaction with high-resolution melting analysis
RNA	Ribonucleic acid
rRNA	Ribosomal ribonucleic acid
SCFA	Short-chain fatty acid
VP2	Viral protein 2
WSAVA	World Small Animal Veterinary Association
